# Odor Sensitivity After Intranasal Insulin Application Is Modulated by Gender

**DOI:** 10.3389/fendo.2018.00580

**Published:** 2018-10-02

**Authors:** Rea Rodriguez-Raecke, Yvonne F. Brünner, Anja Kofoet, Smiljana Mutic, Christian Benedict, Jessica Freiherr

**Affiliations:** ^1^Diagnostic and Interventional Neuroradiology, University Hospital, RWTH Aachen University, Aachen, Germany; ^2^Sensory Analytics, Fraunhofer Institute for Process Engineering and Packaging IVV, Freising, Germany; ^3^Department of Neuroscience, Uppsala University, Uppsala, Sweden

**Keywords:** intranasal insulin, odor sensitivity, olfaction, olfactory threshold, n-butanol, peanut

## Abstract

Obesity constitutes a global health care problem, and often eating habits are to blame. For intervention, a thorough understanding of energy intake and expenditure is needed. In recent years, the pivotal role of insulin in connection to energy intake was established. Olfactory sensitivity may be a target of cerebral insulin action to maintain body weight. With this experiment, we aimed to explore the influence of intranasal insulin on olfactory sensitivity for the odors n-butanol and peanut in a placebo-controlled, double-blind setting in a within-subject design. All subjects participated in two experimental sessions on separate days and received either intranasal insulin or placebo in a pseudorandomized order. Application was followed by two olfactory threshold tests for n-butanol and peanut in a pseudorandomized order. After a single dose of intranasal insulin (40 IU) or placebo (0.4 ml), olfactory sensitivity for the odorants n-butanol and peanut were examined in 30 healthy normosmic participants (14 females). Measured blood parameters revealed no decrease in plasma glucose, however, insulin, leptin and cortisol levels were affected following intranasal application. Females' but not males' olfactory sensitivity for n-butanol was lower after intranasal insulin administration vs. placebo. In contrast, olfactory sensitivity for peanut was not influenced by intranasal insulin application. Our results indicate that the effects of cortical insulin levels on processing of specific odors is likely modulated by gender, as central increase of insulin concentration led to a reduced olfactory sensitivity for n-butanol in women only, which might be due to differentially regulated insulin and leptin signaling in men and women.

## Introduction

In the context of our modern way of life and nutrition, diabetes, and obesity, as well as their serious sequelae, are among the most important health risks of the twenty-first century. Among the multiple contributing factors connected to food intake and energy expenditure, the influence of the chemical senses and their understanding are crucial. The olfactory system is considered to be tightly intertwined with the endocrine system in the regulation of chemical state and nutritional need and was suggested to have a secondary function as an internal nutritional sensor (1). One of the most crucial factors might be insulin signaling. A study using slices of the olfactory bulb in an animal model established that insulin can alter both spontaneous and olfactory nerve-induced firing activities and thus could impact odor detection (2).

The human brain is an insulin-sensitive organ and presents an accumulation of insulin receptors in the hypothalamus, the olfactory bulb, and the hippocampus (3–5). Those areas fulfill crucial tasks during the processing of olfactory information (6). Insulin receptors are not only found in those olfactory processing sites, but also in the olfactory epithelium (1). Thus, intranasal insulin might influence olfactory processing either on a peripheral level at the olfactory receptors or on the level of central olfactory processing. The ability of substances (e.g., insulin) to enter the cerebrospinal fluid from the nasal mucosa has been observed in various studies (7–12).

Two previous studies with healthy participants indicated that an increased insulin level in the cerebrospinal fluid (CSF) leads to a decrease of olfactory sensitivity for the odorant n-butanol (13, 14). In the first study by Ketterer et al. (13), the hyperinsulinemic clamp technique, a highly invasive method during which plasma insulin concentration is acutely raised while peripheral glucose levels are held constant at euglycemic levels, was used. In the second study presented by our group (14), a non-invasive technique, the intranasal insulin administration, was utilized. This method effectively delivers insulin to the central nervous system (CNS) in the absence of relevant systemic absorption (9) and may allow a selective examination of insulin effects on the human brain avoiding side effects in the body periphery. Both studies included men and women and did not investigate sex-specific subgroups, as this would have resulted in insufficient sample sizes.

Besides those effects of intranasal insulin on olfactory processing, there is compelling evidence that an increase of cerebral insulin levels has an impact on the mediation of satiety leading to a reduction of snack size (15–18). Insulin as endogenous satiety signal suggests an anorexic effect of intranasal insulin and hints towards an involvement of cerebral insulin in the regulation of food intake. It can be considered a metabolic key signal in the homeostatic mediation of satiety (19). More specifically, Hallschmid et al. reported that regular treatments with central insulin reduced body weight in men but not in women (20). This sex-specific differential sensitivity might be crucial for insulin modulation on the olfactory system as well. The anorexic effect of intranasal insulin is likely to be transmitted via a manipulation of the chemosensory signaling cascade on a peripheral receptor level or a central processing level. Since insulin is such a crucial metabolic hormone during the mediation of satiety, it is possibly associated with altered olfactory sensitivity. Since the effects of intranasal insulin on energy homeostasis are sex-specific (20, 21), sex effects related to odor detection are probably relevant as well. As a gold standard for investigating olfactory thresholds in humans, the Sniffin' Sticks with the odor threshold, discrimination, and identification (TDI) score (22) are commonly applied for assessment of olfactory function in healthy participants and patients suffering from various diseases (23–26). Research investigating olfactory sensitivity after intranasal insulin application is scarce, nonetheless in patients with hyposmia, intranasal insulin is reported to increase odor detection sensitivity for n-butanol (27, 28) while in healthy participants the performance is reduced (13, 14). In all experiments, sex-specific effects were set aside by investigating mixed samples of men and women, and in all studies, n-butanol was used.

With regards to olfactory function, women often outperform men (29), but regarding weight loss after insulin administration, only men were susceptible to this effect (20). Based on this incoherent landscape of current knowledge on intranasal insulin and the chemical senses, it appears plausible to explore not only the effects of intranasal insulin on olfaction in both sexes, but also on olfactory sensitivity to n-butanol in comparison to other olfactory stimuli. Olfactory threshold tests with an alternative odor than n-buntanol are rarely used. To our knowledge, no study evaluated olfactory detection thresholds after intranasal insulin application with an alternative odor to n-butanol, nor with a focus on sex-specific effects.

With our current study, we aimed to investigate the effects of intranasal insulin (40 IU) on sensitivity for the odors n-butanol and peanut in healthy men and women. With this follow-up study, we intend to evaluate insulin effects on test performance for n-butanol in a larger cohort and additionally address possible intranasal insulin effects for the odor peanut as well as sex-specific differences regarding the insulin effects.

## Materials and methods

### Participants and preliminary screening

This study was carried out in accordance with the recommendations of local ethics review board. The protocol was approved by the local ethics review board. All subjects gave written informed consent in accordance with the Declaration of Helsinki.

Only healthy participants were included. During the timeframe of the study, none of the participants was taking medication or showed any abuse of substances. All subjects had normal weight, were non-smokers and were instructed not to wear perfume or get in contact with products that have a strong smell during experimental sessions. All females used oral contraceptives but no other medication. In our preliminary screening session, all participants underwent a laboratory screening of standard clinical blood parameters (Gamma-GT, creatinine, TSH, LDL, HDL, triglyceride, leptin, glucose, insulin, and cortisol) and the Body Mass Index (BMI) was calculated to ensure general healthiness. Based on the preliminary screening session, five participants with abnormal blood levels were excluded, resulting in a final sample size of 30 normosmic subjects (16 males/14 females; age: *M* = 24.63, *SEM* = 3.61 years; BMI: *M* = 21.93, *SEM* = 1.69 kg/m2). Further, subjects completed three clinical questionnaires for general psychopathology, depression, and cognitive function [SCID I—Structured Clinical Inventory for DSM-IV Axis I Disorder, BDI—Beck Depression Inventory II; BDI cut-off ≥ 9; females: *M* = 1.21, *SEM* = 0.39; males: *M* = 2.43, *SEM* = 0.75; *t*_(28)_ = 1.388, *p* = 0.176, MoCA—Montreal Cognitive Assessment] (30–32). Females demonstrated a higher performance during the MoCA test [cut-off ≥ 26; females: *M* = 28.86, *SEM* = 0.23; males: *M* = 27.63, *SEM* = 0.28; *t*_(28)_ = −3.282, *p* = 0.003]. Females and males did not differ regarding age [*t*_(28)_ = 1.135, *p* = 2.67] and BMI [*t*_(28)_ = 1.582, *p* = 0.125] (see Table 1). The extended version of the Sniffin' Sticks identification test [MONEX-40 (33)] was used as a screening tool to categorize the subjects as normosmic. None of the subjects scored less than the cut-off value of 27 in the MONEX-40. All participants were considered normosmic and included in the experimental procedures.

**Table 1:** Preliminary screening parameter for BDI, BMI, and age (*n* = 30 subjects, males = 16, females = 14).

**Screening parameter**	**Cut-off value**	**Sex**	**Mean**	**SEM**
BDI	>9	Males	2.43	0.75
		Females	1.21	0.39
BMI	>24.99	Males	22.37	0.43
		Females	21.42	0.42
Age	–	Males	25.31	1.05
		Females	23.86	0.73

*Females and males did not differ regarding BDI, BMI, and age. Data are provided as means and standard error of means*.

### Experimental setting

**Figure 1:**
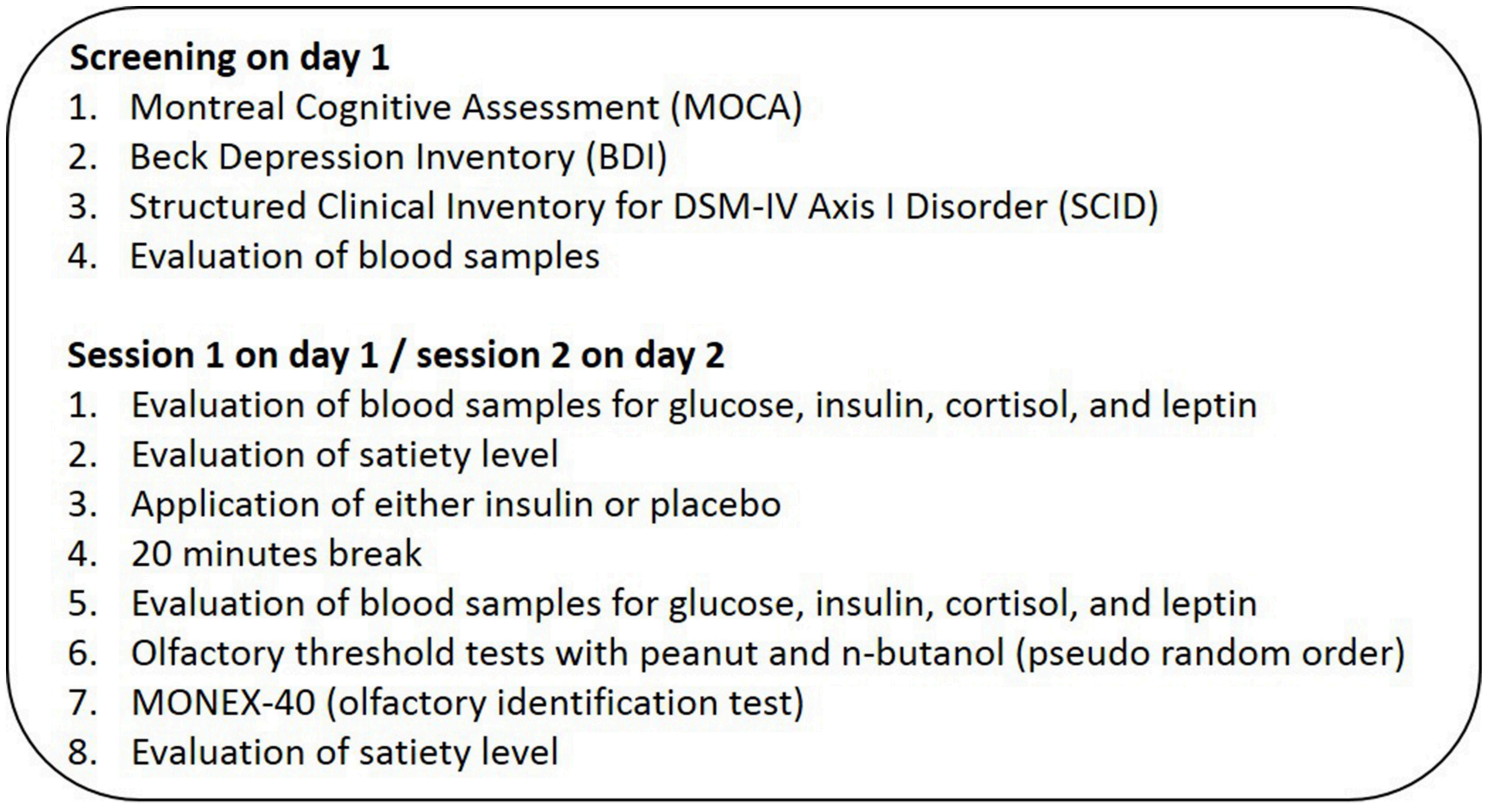
Study design. Each subject was measured twice in the morning after 12 h of overnight fasting. In the beginning and at the end subjects answered a questionnaire about their satiety status. Following the questionnaire, 30 normal-weight, and normosmic participants got either an intranasal application of insulin (40 IU) or placebo solution (0.4 ml) and performed two threshold tests, one with n-butanol and one with peanut odor. Blood samples were taken before and 20 min after intranasal administration. Each session ended with the MONEX-40 identification test.

After the preliminary screening session, all 30 subjects participated in two experimental sessions on separate days during which they received either an intranasal insulin application (40 IU, human insulin, Novo Nordisk Pharma GmbH) or a placebo solution (0.4 ml, Novo Nordisk Pharma GmbH) in a pseudorandomized order (for a detailed overview of experimental protocol illustration see Figure 1). Experimenter and participants were blinded regarding the applied substance. The nasal spray solution containing insulin or placebo (spray bottles, Aero Pump GmbH, Germany) contained the same liquid solvent, which causes a mild irritation in the nose for a few minutes; the only difference between the treatment conditions was that the placebo solution did not contain insulin. In an independent pilot study in our lab, 12 normosmic participants evaluated the odor quality of the insulin and placebo solution as perceptually similar (14), and a bias arising from the chemical solvent can be ruled out. The experimental sessions started in the morning at 08:00 a.m. after a 12 h overnight fast and the second session was conducted within the following 1–8 days (*M* = 2.5, *SEM* = 0.35 days) after the first session. During each experimental session, all subjects completed ratings regarding their current satiety status on a 100-mm visual analog scale (0 = not hungry/not craving for food/stomach feels empty, 100 = very hungry/strong craving for food/stomach feels full). The first rating was given before intranasal application and the second at the end of each session. Following the first satiety rating, blood parameters glucose, insulin, leptin, and cortisol were taken before and 20 min after insulin or placebo administration (9). For the intranasal application, the participant was instructed to lean back in the chair, lift the chin and the study experimenter sprayed the substance into the subject's nostrils, 2 puffs each nostril. This was followed by a 20 min break for expected substance delivery from the nose to the brain (9). One session lasted 50 min and an increased level of cerebral insulin is probably maintained for at least 60 min (9). Subjects performed two olfactory threshold tests with the odorants n-butanol and peanut in a pseudorandomized and counterbalanced study design. For the n-butanol olfactory threshold test, a standardized test of the Sniffin' Sticks (Burghart, Medizintechnik GmbH, Germany) (22) was used. For the peanut olfactory threshold test, a custom-made test with glasses containing 16 dilution steps with concentrations ranging from 0.0029 to 17.53% of peanut oil (Takasago “Natural & Artificial Peanut” Flavor OS) in diethylphthalate (Sigma-Aldrich, Germany) was used. During both threshold tests subjects were blindfolded. Subjects completed the odor identification test MONEX-40 with subjective ratings of pleasantness and intensity on 100-mm visual analog scales (0 = very unpleasant/low intensity, 100 = very pleasant/high intensity). To control for effects of olfactory adaptation, the extended MONEX-40 test was conducted after each threshold test. We further checked if olfactory identification ability as well as intensity and hedonic evaluation of the odors changed due to intranasal insulin application.

### Statistical analyses

All data were analyzed using SPSS software (IBM SPSS Statistics 22.0, Chicago, US). A repeated-measures ANOVA for each threshold test with the within-subject factor “treatment” (insulin vs. placebo), the between-subject factor “sex” (women vs. men) and “application order” (insulin first vs. placebo first) was computed. For analyzing blood parameters, repeated-measures ANOVAs including the within-subject factor “treatment” (insulin vs. placebo), “time” (before vs. after) as well as the between-subject factor “sex” (women vs. men) and “application order” (insulin first vs. placebo first) were calculated. Partial eta-squared (η^P2^) was used as an index of effect size. Subsequent *post-hoc* tests with Bonferroni correction were computed to further inspect the comparisons. Additionally, repeated-measures ANOVAs with the within-subject factor “treatment” and the between-subject factor “sex” were utilized for the analysis of the satiety questionnaires and MONEX-40 identification data with pleasantness and intensity ratings. All data are presented as means (M) and standard error of means (SEM). A *p* < 0.05 was considered significant.

### Data availability

The datasets generated and analyzed during the current study are available from the corresponding authors, without undue reservation, on reasonable request.

## Results

### Olfactory tests

**Figure 2:**
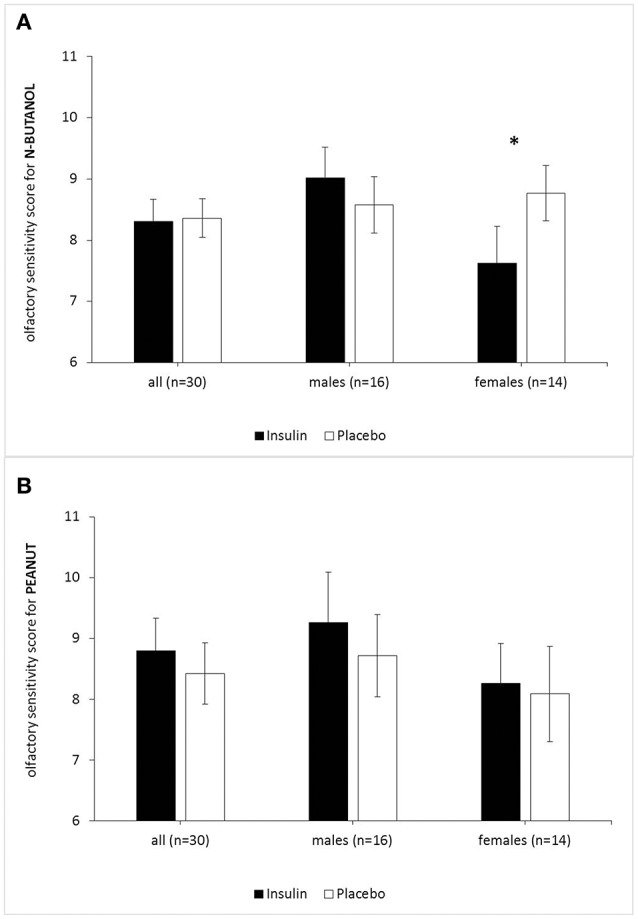
Acute effects of intranasal insulin (40 IU, black bars) and placebo (0.4 ml, white bars) on olfactory sensitivity (highest olfactory sensitivity score = 16) for the odorants peanut **(A)** and n-butanol **(B)**. *n* = 30 (males *n* = 16, females *n* = 14). While females showed reduced olfactory sensitivity for n-butanol (*p* = 0.045) after intranasal insulin, males did not. Olfactory sensitivity for peanut was not influenced by intranasal insulin in both genders. Data are provided as means and SEMs; *p < 0.05.

The two-way repeated-measures ANOVA for n-butanol threshold testing showed no main effect of treatment (insulin vs. placebo), but a significant interaction effect of treatment^*^sex [*F*_(1, 28)_ = 4.49, *p* = 0.043, η^P2^ = 0.138]. Paired comparisons showed, that only in female subjects olfactory sensitivity for n-butanol was significantly reduced after intranasal insulin compared to the placebo application [insulin: *M* = 7.63, *SEM* = 0.60, placebo: *M* = 8.77, *SEM* = 0.45; *F*_(1, 28)_ = 4.41, *p* = 0.045, η^P2^ = 0.136]. Male subjects showed no effect of treatment for the odorant n-butanol [*F*_(1, 28)_ = 0.738, *p* = 0.400] (Figure 2A). No main and no interaction effects were found for the odorant peanut (Figure 2B). As no effect of the between-subject factor “application order” was established for any of our threshold tests, we excluded this parameter from further analyses.

### Blood parameter analysis of glucose, insulin, leptin, and cortisol levels

**Figure 3:**
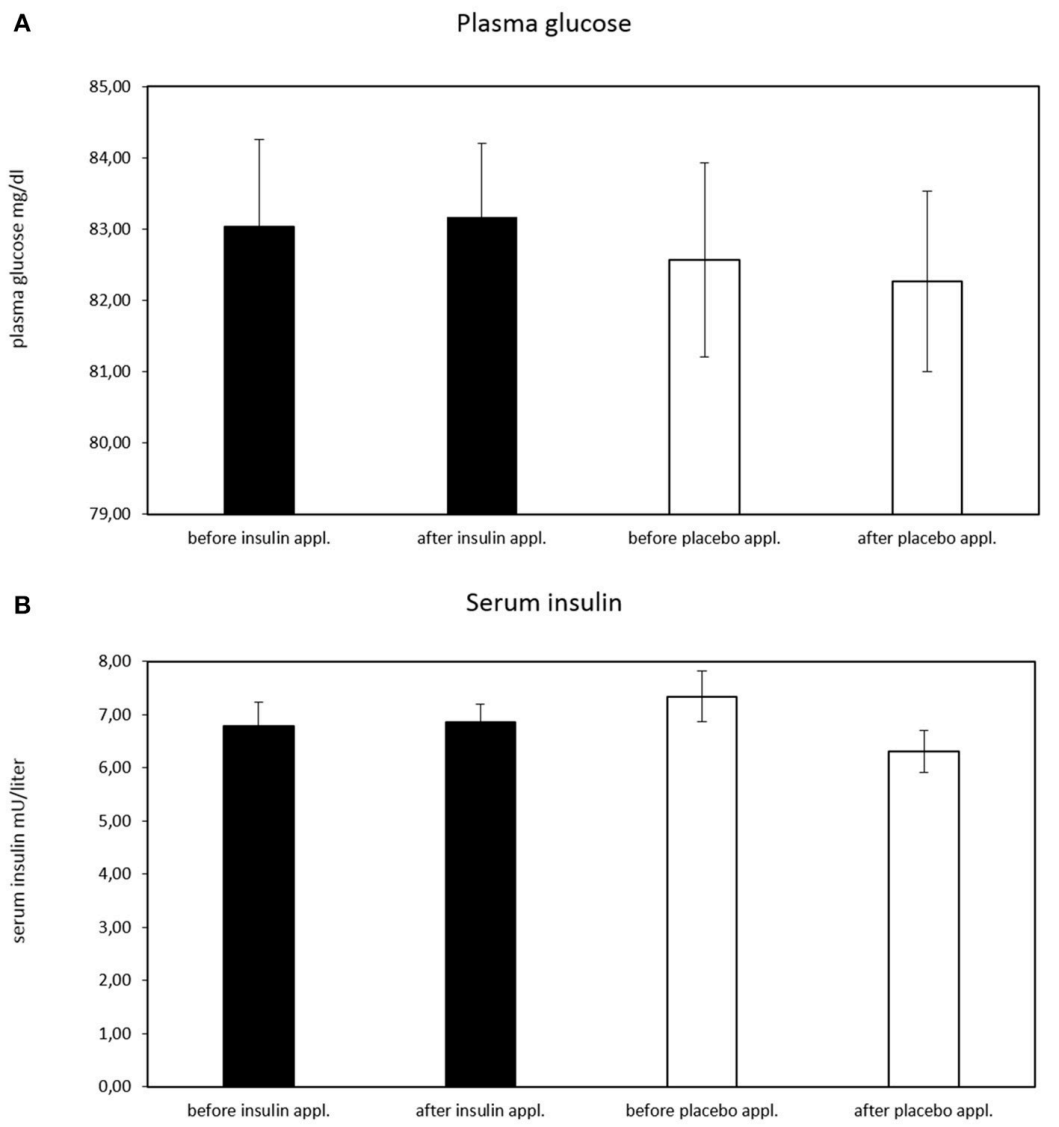
Plasma glucose concentration before and after intranasal insulin and placebo for *n* = 30 subjects. **(A)** No effect of intranasal application on circulating plasma glucose level is evident, **(B)** significant treatment*time interaction in serum insulin levels [*F*_(1, 28)_ = 5.01, *p* = 0.033, η^P2^ = 0.152; before insulin *M* = 6.813, *SD* = 0.444; after insulin *M* = 6.872, *SD* = 0.341; before placebo *M* = 7.385, *SD* = 0.472; after placebo *M* = 6.373, *SD* = 0.364). The paired comparisons revealed no significant results (all *p* > 0.205).

As expected, no effect of the intranasal application on circulating plasma glucose level (*p* ≥ 0.494 for all ANOVA effects) was established (Figure 3A). There was a significant treatment^*^time interaction in serum insulin levels [*F*_(1, 28)_ = 5.01, *p* = 0.033, η^P2^ = 0.152] with lower insulin levels after placebo treatment (before insulin treatment *M* = 6.813, *SD* = 0.444; after insulin treatment *M* = 6.872, *SD* = 0.341; before placebo treatment *M* = 7.385, *SD* = 0.472; after placebo treatment *M* = 6.373, *SD* = 0.364). The paired comparisons revealed no significant results (all *p* > 0.205) (Figure 3B).

Leptin values of one subject could not be analyzed, thus the results of leptin blood levels are based on a group of 29 subjects. There was a significant main effect of time for serum leptin level [*F*_(1, 27)_ = 7.92, *p* = 0.009, η^P2^ = 0.227] with a lower serum level in the second blood sampling (before: *M* = 5.60, *SD* = 0.620; after: *M* = 5.01, *SD* = 0.555). However, no interaction effect treatment^*^time was found in serum leptin levels [*F*_(1, 27)_ = 0.011, *p* = 0.919]. For cortisol levels a significant main effect of time for the serum cortisol level emerged [*F*_(1, 28)_ = 60.43, *p* < 0.001, η^P2^ = 0.683] also with a lower serum cortisol level in the second blood sampling (before: *M* = 25.32, *SD* = 0.869; after: *M* = 23.14, *SD* = 0.854). Further, no interaction effect treatment^*^time was established for serum cortisol [*F*_(1, 28)_ = 8.30, *p* = 0.370]. To rule out any modulation effect of the lower serum leptin and cortisol level on olfactory sensitivity for the odorant n-butanol, *post-hoc* Pearson's correlations between post-treatment serum leptin and cortisol level with olfactory sensitivity score for n-butanol in the insulin condition were computed but did not yield significant results (leptin: *r*_29_ = −0.191, *p* = .321; cortisol: *r*_30_ = −0.180, *p* = 0.340). Additionally, we also controlled for the contribution of leptin and cortisol levels using the blood levels as covariates in the repeated-measures ANOVAs on olfactory performance and established that this did not change the results reported above. In the calculated repeated-measures ANOVAs for the blood levels of glucose, insulin, leptin, and cortisol no sex effects were found.

### Satiety questionnaires

The results of the satiety questionnaires showed a main effect of time, independently of treatment for level of hunger [*F*_(1, 28)_ = 5.624, *p* = 0.025, η^P2^ = 0.167] and food craving [*F*_(1, 28)_ = 12.643, *p* ≤ 0.001, η^P2^ = 0.311]. Participants were less hungry (before: *M* = 5.38, *SEM* = 0.40; after: *M* = 6.32, *SEM* = 0.30, and felt less craving after the experimental session (before: *M* = 4.66, *SEM* = 0.42; after: *M* = 6.14, *SEM* = 0.34). There was no effect for fullness of stomach. We found no sex effects. Further, there was no correlation between post-treatment serum leptin levels and the satiety questionnaires with intranasal insulin application (hungry: *r*_29_ = 0.028, *p* = 0.887; craving: *r*_29_ = −0.088, *p* = 0.651].

### MONEX-40 identification test

The results of the MONEX-40 identification test extended by hedonic odor ratings of pleasantness and intensity remained unaffected in response to intranasal insulin (all *p* ≥ 0.349). All subjects were normosmic and neither olfactory identification capability [insulin: *M* = 32.92, *SEM* = 0.449; placebo: *M* = 32.91, *SEM* = 0.389, *F*_(1, 28)_ = 0.002, *p* = 0.969, η^P2^ < 0.001] nor hedonic ratings of pleasantness [insulin: *M* = 64.64, *SEM* = 1.77; placebo: *M* = 64.85, *SEM* = 1.50; *F*_(1, 28)_ = 0.051, *p* = 0.822] and intensity [insulin: *M* = 74.97, *SEM* = 2.35; placebo: *M* = 75.65, *SEM* = 2.15; *F*_(1, 28)_ = 0.278, *p* = 0.602] differed between treatments. Females and males did not differ concerning the MONEX-40 score and the pleasantness and intensity ratings.

## Discussion

In this double-blind crossover study, we explored the effects of intranasal insulin on human olfactory sensitivity for the odorants n-butanol and peanut. The decision to use a single dose of insulin administration (40 IU) was based upon our preceding work in which we already demonstrated an olfactory sensitivity reduction for n-butanol in 17 healthy participants (14). Since intranasal insulin might mediate satiety, we hypothesized a reduced olfactory sensitivity for the odors n-butanol and peanut, as well as a reduced pleasantness and intensity rating during the MONEX-40 identification test.

Blood levels of insulin, glucose, leptin, and cortisol were monitored before and 20 min after intranasal insulin and placebo administration. This was necessary to prevent a relevant influence of intranasal insulin application on peripheral insulin and blood glucose level but also on neuronal circuit's activation for the endogenous glucose production (20, 34). Since glucose homeostasis is also regulated by leptin, causing effects on the glucose-insulin metabolism (35), leptin blood levels were measured as well. Also, cortisol level was measured, because it is involved in carbohydrate metabolism and might be secreted due to low blood glucose levels. To be able to assume that intranasal insulin acts centrally, we did not expect relevant influences of intranasal insulin application on peripheral glucose and insulin levels, however, we expected time effects for leptin and cortisol, independently of insulin or placebo application, possibly caused by an 12 h overnight fast of our subjects.

Olfactory sensitivity for n-butanol in the whole group of subjects was not decreased. However, this olfactory sensitivity decrease for the odorant n-butanol emerged when looking at the subgroup of female subjects only. Olfactory sensitivity for the odorant peanut and the hedonic ratings of pleasantness and intensity remained unaffected by intranasal insulin in both sexes.

Concerning the subgroup of females, we were able to demonstrate again (14) that a single dose of intranasal insulin (40 IU) reduces olfactory sensitivity for n-butanol in contrast to a placebo solution, but we failed to show this effect for the whole group of subjects. Interestingly, while our previous research did not yield significant sex differences of olfactory sensitivity for the odorant n-butanol following intranasal insulin application (14), we did observe sex differences in the current study with a larger cohort. Taking a closer look at our previous study, a reason for this inconsistency might be the low number and unequal distribution of females and males. However, when we pooled the data of the current study with the data from our previous study (14), the result of decreased olfactory sensitivity for n-butanol in females is distinctly stable (both studies: *n* = 47, females = 21, males = 26). Interestingly, an animal study reported that male rats decreased food intake with increased cerebral insulin for 24 h, while female rats did not show this effect, but displayed greater effects with leptin (36). Probably a complex interaction of insulin, leptin and other factors regulates a balanced energy consumption. Recent evidence in research shows a reduction of highly palatable food intake in women after applying postprandial intranasal insulin of 160 IU (15). This data indicates that enhanced brain insulin levels play a tentative role during the postprandial phase. One remarkable difference concerning the previously described studies in comparison to our experiment is that in those studies intranasal insulin application in relation to real food intake was tested, which may contribute to increased meal-related effects in the tested subjects (15, 21). Since serum cortisol levels in humans have been correlated to olfactory sensitivity previously (37) and leptin has effects on the glucose-insulin metabolism (35, 38), cortisol and leptin blood levels were measured in our study. However, we did not find significant correlations between post-treatment serum leptin and cortisol levels as well as olfactory threshold score. Neither did *post-hoc* tests for serum insulin show any correlations in our study. This suggests no direct link between serum blood parameters of leptin or cortisol and decreased olfactory sensitivity for n-butanol in females. Notably, in our current study participants showed no sex difference regarding BMI, age and olfactory identification performance (MONEX-40). Only in respect to cognitive performance (MoCA), females performed significantly better. Whether this is linked to the reduced olfactory sensitivity for n-butanol remains unclear, as we did not address this topic and cannot provide an explanation for this result. Olfactory sensitivity reflects the functionality of the peripheral olfactory system (6) and cognitive performance should have only minor influences on olfactory sensitivity estimation. Contrarily to our expectations, a possible anorexic effect of intranasal insulin on sensitivity to the odor peanut did not occur. Possibly, peanut odor is not representing an odorant sufficiently distinctive as food-related. We would restrain from transferring our results of olfactory sensitivity to further non-food and food odorants. There also seems to be a high interindividual variability of test performance. Furthermore, the inconsistent results for peanut and n-butanol sensitivity might be in association with their chemical compositions. N-butanol is a single compound, whereas peanut is a mixture comprised of multiple chemical compounds. Further studies should aim to elucidate differences regarding insulin effects on single molecule vs. multiple molecules odorants to further investigate the specificity of centrally acting insulin in olfaction (39). Albeit, we cannot explain those results based on the current data and would need more studies using different odorants to get deeper insights. Another explanation could be that our dose of 40 IU of intranasal insulin was too low to modulate food-related brain areas compared to previous human studies with a dose of 160 IU of insulin (21). Nevertheless, our experimental design using 40 IU insulin was based on previous experiments, in which we already demonstrated insulin effects on human olfactory sensitivity (14).

Finally, our subjects felt less hungry and craved less for food, an effect that was not affected by treatments of intranasal applied insulin or placebo. An explanation could be that the subjects were distracted by the olfactory tests and paid less attention to their physical state during the second evaluation (40).

As insulin receptors are found in the olfactory epithelium (1), this is where first odor detection takes place. Nevertheless, since higher cognitive processes are necessary during odor sensing, it is likely that enhanced cerebral insulin signaling is associated with the effects of intranasal insulin on olfactory perception in our study. The spectrum of enhanced brain insulin activity and its influence on body energy metabolism is poorly understood. Possibly, females' and males' different responses on cerebral insulin signaling are associated with a biphasic response of central insulin on the peripheral insulin metabolism (41). Born et al. (9) revealed the biphasic course of insulin concentration in the cerebrospinal fluid, consisting of a first peak 10 min and a second peak 30 min after intranasal insulin application. We would like to encourage further research to examine the effect of stimulus complexity regarding chemical compounds and timing of insulin release. Sex differences regarding the effects of intranasal applied insulin should be further investigated using odor stimuli that signal edibility.

In conclusion, intranasal insulin application led to a reduced olfactory sensitivity for the odorant n-butanol in females but not in males. Naturally, the modulation of olfactory performance by insulin in healthy subjects only reflects a section of the complexity of human energy homeostasis. However, insulin was shown to withhold a prominent role in the interaction of the CNS with whole-body energy metabolism, which should be taken into account in future studies related to the pathophysiology of reduced insulin levels (insulin resistance) and insulin activity in the CNS in patients with type 2 diabetes or in patients with obesity (42, 43).

## Author contributions

RR-R wrote the manuscript, interpreted results, had full access to all data in the study, and takes the responsibility for the integrity and accuracy of data analysis. YB analyzed the data and revised the manuscript. AK acquired the data. SM and CB interpreted the results and revised the manuscript. JF is the guarantor of this work, supervised the study, interpreted results and revised the manuscript.

### Conflict of interest statement

The authors declare that the research was conducted in the absence of any commercial or financial relationships that could be construed as a potential conflict of interest.
